# Are all types of empathy associated with lower aggression in athletes? A cross-sectional study on Iranian athletes

**DOI:** 10.1186/s40359-022-00985-4

**Published:** 2022-11-22

**Authors:** Elham Mahmoudi, Amin Nakhostin-Ansari, Maryam Ranjbar, Amir Hossein Memari

**Affiliations:** 1 Sports Medicine Research Center, Neuroscience Institute, Tehran University of Medical Sciences, Tehran, Iran; 2 Neuromusculoskeletal Research Center, Iran University of Medical Sciences, Tehran, Iran; 3 Department of Psychology, University of Tehran, Tehran, Iran

**Keywords:** Aggression, Athletes, Empathy, Sports

## Abstract

**Background:**

This study aimed to evaluate the association between cognitive and affective empathy and aggression in a sample of Iranian athletes.

**Methods:**

We designed a cross-sectional study. The participants were selected by multistage random sampling among six colleges in Tehran, Iran’s capital. We used the interpersonal reactivity index (IRI) to evaluate empathy, and Reactive Proactive Aggression Questionnaire, and the Buss–Perry aggression questionnaire to evaluate aggression.

**Results:**

In total, 492 athletes with a mean age of 27.42 years (SD = 7.72) participated in the study, of which 298 (60.6%) were male, and 194 (39.4%) were female. IRI’s fantasy and personal distress subscales scores were positively associated with proactive and reactive aggression scores (*p* < 0.05). The score of the perspective-taking subscale of IRI was negatively associated with proactive and reactive aggression scores (*p* < 0.05). The score of the empathic concern subscale of IRI had a negative association with the proactive aggression score (*p* < 0.001). The score of the perspective-taking subscale of IRI had negative associations with all Buss–Perry aggression questionnaire subscales’ scores (*p* < 0.05). The score of the personal distress subscale of IRI had positive associations with all Buss–Perry aggression questionnaire subscales’ scores (*p* < 0.05), except with the verbal aggression subscale score. The score of the fantasy subscale of IRI was positively associated with the score of the hostility subscale of the Buss–Perry questionnaire (*p* = 0.001).

**Conclusion:**

Perspective-taking is negatively associated with all kinds of aggression in athletes. Future studies can be conducted to determine the possible role of perspective-taking in preventive aggression, which can be a target for interventions. On the other hand, the score of the personal distress subscale of IRI is positively associated with all types of aggression scores, indicating that not all types of empathy inhibit aggression in athletes.

## Introduction

Physical and verbal aggressions are common behaviors among athletes, especially in contact sports [1–3]. Aggression is defined by Baron et al. as “any form of behavior directed toward the goal of harming or injuring another living being who is motivated to avoid such treatment [4].” Aggressive behaviors are categorized into two subtypes. The first subtype is called reactive or hostile aggression. Reactive aggression is an impulsive or emotionally aggressive response to a minimal provocation that has emerged as a loss of behavioral control. The second subtype is called proactive or instrumental aggression [5]. In contrast, proactive aggression is the planned, instrumental behavior involving computed efforts to get important resources [5]. However, other terminologies beyond reactive and proactive aggression have been utilized to describe aggression in the sport and non-sport contexts. In sports, aggression is commonly divided into sanctioned and unsanctioned types [6]. In brief, sanctioned aggressions are not out of the game’s laws and roles, contrasting the unsanctioned aggression [7]. Unsanctioned aggression may lead to physical and psychological consequences, and there are various motivations for unsanctioned aggression, such as anger, power, and thrill [6, 8].

Aggression in the sports context has been the subject of studies for years. The roles of various factors, such as gender, the importance of the game, the type of sport, and empathy in athletes’ aggression, have been evaluated in previous studies [9–12]. There are some controversies on the role of aggression in athletes’ performance. It is suggested that hostile aggression may improve the athletes’ performance by raising their arousal to optimal levels [13, 14]. Also, physicality and authorized aggression were perceived as positive mechanisms in certain women’s sports because there is a myth about aggression and anxiety will increase athletic performance [7]. Although it seems true sometimes, studies have shown that aggression does not facilitate performance. For example, aggression increases people’s arousal level and the focused attention to the results of non-executive cues. (e.g., hurting the opponent) that could reduce or interfere with the performance [15, 16].

Studies have shown that aggression has increased among athletes in the past decades. The rise in aggressive behaviors could have resulted from anger rumination, provocation, and the use of anabolic steroids [17–20]. Several studies on athletes’ aggression and its mediating factors have been conducted. It is found that aggressive behavior could have serious consequences, such as injury, thus enhancing the risk of further damage [21]. In addition to the effects of aggression on performance, aggressive behavior could lead to the players’ exclusion or legal consequences [22]. The International Society of Sport Psychology (ISSP) has some recommendations to reduce aggression in sports, such as considering serious punishments for violating roles by authorities and coaches, participation of athletes in programs designed to reduce aggression tendencies, and emphasizing fair play from junior levels [23]. Therefore, it is crucial to examine mediator factors of aggression to design interventions to regulate athletes’ aggression.

One of the factors related to aggressive behavior is empathy. Studies showed that empathic responses negatively correlated with prosocial and/or antisocial behavior [24, 25]. A person with high empathy could understand others’ feelings via perspective-taking by imagining how the other person feels [26]. Some researchers have intended two components for empathy: cognitive empathy, understanding others’ emotions, and affective empathy, an emotional response, such as verbal or facial, to others’ emotions [27, 28]. It is shown that different anatomical sites of the brain are responsible for cognitive and affective empathy [29]. People with less cognitive empathy have less ability to tolerate opposite viewpoints, leading to aggressive behaviors [30]. Also, individuals with low affective empathy could not understand others’ suffering, the pain inflicted, and the fear of the victim [31]. Conversely, empathy enhancement has inhibited aggressive acts in a bid to reduce their emotional distress. Hence, empathy’s cognitive and affective components seem to inhibit aggression [26]. It should be noted that cognitive and affective empathy may have different roles in preventing aggressive behavior [32]. It is shown that high levels of affective empathy in girls can reduce cyberbullying, regardless of cognitive empathy [33]. However, roles of affective and cognitive empathy in preventing aggression in athletes has not been evaluated yet.

Association between empathy and aggression has been studied in athletes [11, 26] and non-athlete [34, 35] populations, which have found inhibitory effects of empathy on aggression prevention. Previous findings are related to athletes from specific countries, and there is little data on athletes with different backgrounds, especially in developing countries [36]. It gets more important as ethnicity is one of the factors affecting individuals’ normative beliefs, which can moderate the association between empathy and aggression [37, 38]. Also, none of the previous studies separately evaluated cognitive and affective empathy’s role in aggressive behavior. Therefore, this study aimed to evaluate the association between cognitive and affective empathy on aggression in a sample of Iranian athletes. Determining the possible association between aggression and empathy among athletes in developing countries with different backgrounds and ethnicities than athletes in developed countries can enhance our knowledge in this regard. We hypothesized that all kinds of empathy have a negative correlation with aggression among athletes.

## Materials and methods

We designed a cross-sectional study. The study protocol was according to the declaration of Helsinki. The ethical committee of the Tehran University of Medical Sciences approved the study protocol (code: IR.TUMS.NI.REC.1399.056).

### Participants

We defined an athlete as someone who is recently registered in a sports team, trained with the team, and participated in sports competitions. Sports were also classified as contact and non-contact. All the participants were selected by multistage random sampling among six colleges and twenty-four classes in Tehran, Iran’s capital. In each college, four classes were randomly selected, and 700 athletes were randomly selected from the students of the included classes. We provided a complete explanation of the study goals and objectives, and those interested were enrolled in the study. All the participants filled out their consent forms. We sent the questionnaires to 700 athletes after they gave informed consent to participate in the study and asked them to complete the questionnaires independently. In total, 492 athletes with a mean age of 27.42 years (SD = 7.72) participated in the study, of which 298 (60.6%) were male, and 194 (39.4%) were female. One hundred and ninety-seven (40%) of the participants were married, and 347 (70.5%) had a university degree (Table 1). Also, 344 (69.9%) were contact sports athletes, and 87 (17.7%) team sport athletes (Table 2).

**Table 1:** Empathy and aggression scores across demographic groups

	Total	Gender			Age group					Marital status			Educational		
		Male (N = 298)	Female (N = 194)	*p* value (Mann–Whitney test)	17–20 years (N = 103)	21–25 years (N = 131)	26–30 years (N = 121)	> 30 years (N = 37)	*p* value (Kruskal– Wallis test)	Married (N = 197)	Unmarried (N = 293)	*p* value (Mann–Whitney test)	Diploma or lower (N = 143)	University education (N = 347)	*p* value (Mann–Whitney test)
Personal distress	13.33 (5.25)	11.92 (4.82)	15.52 (5.15)	< **0.001**	12.68 (5.36)	12.84 (5.66)	13.98 (5.27)	13.78 (4.68)	> 0.05	13.93 (5.21)	12.96 (5.26)	> 0.05	13.32 (5.61)	13.36 (5.11)	> 0.05
Empathic concern	19.81 (4.51)	19.11 (4.73)	20.95 (3.95)	< **0.001**	19.43 (4.51)	18.62 (4.5)	20.35 (4.24)	20.85 (4.53)	< **0.001**	20.23 (4.44)	19.57 (4.56)	> 0.05	19.57 (4.65)	19.94 (4.47)	> 0.05
Fantasy	14.34 (5.54)	13.56 (5.06)	15.66 (5.92)	< **0.001**	15.1 (5.16)	14.22 (5.38)	14.43 (5.66)	13.99 (5.76)	> 0.05	14.03 (5.64)	14.63 (5.42)	> 0.05	13.93 (5.23)	14.58 (5.62)	> 0.05
Perspective-taking	15.79 (4.48)	15.64 (4.44)	15.98 (4.53)	> 0.05	15.19 (4.67)	15.49 (4.46)	15.36 (4.4)	16.85 (4.27)	< **0.05**	16.18 (4.31)	15.51 (4.57)	> 0.05	15.4 (4.5)	15.93 (4.46)	> 0.05
Interpersonal reactivity index	63.36 (11.37)	60.23 (10.32)	68.28 (11.15)	< **0.001**	62.15 (11.36)	61.53 (10.79)	64.27 (11.13)	65.42 (11.77)	< **0.05**	63.62 (11.52)	62.63 (11.19)	> 0.05	62.5 (11.49)	63.79 (11.29)	> 0.05
Reactive aggression	7.19 (3.62)	7.11 (3.71)	7.32 (3.53)	> 0.05	8.45 (3.62)	7.72 (4.29)	6.84 (3.25)	6.09 (2.9)	< **0.001**	6.64 (3.25)	7.57 (3.84)	**0.006**	7.72 (3.85)	6.98 (3.54)	> 0.05
Proactive aggression	2.56 (3.04)	2.94 (3.37)	1.92 (2.32)	**0.002**	3.3 (2.95)	3.23 (4.03)	2.06 (2.31)	1.77 (2.23)	< **0.001**	2.1 (2.55)	2.84 (3.3)	**0.029**	2.98 (3.52)	2.36 (2.8)	> 0.05
Reactive Proactive Aggression Questionnaire	9.73 (5.84)	10.06 (6.18)	9.25 (5.27)	> 0.05	11.76 (5.6)	10.95 (7.44)	8.91 (4.71)	7.87 (4.38)	< **0.001**	8.74 (4.99)	10.41 (6.28)	**0.008**	10.7 (6.48)	9.35 (5.53)	> 0.05
Verbal aggression	12.48 (4.8)	12.71 (4.91)	12.15 (4.69)	> 0.05	13.11 (4.77)	12.4 (4.63)	12.77 (4.91)	11.84 (4.95)	> 0.05	12.02 (4.94)	12.8 (4.73)	< **0.05**	12.16 (4.78)	12.62 (4.85)	> 0.05
Physical aggression	21.62 (6.84)	22.5 (7.02)	20.44 (6.38)	< **0.01**	24.09 (7.31)	22.48 (7)	21.52 (6.54)	19.2 (5.74)	< **0.001**	20.45 (6.25)	22.49 (7.1)	< **0.01**	21.87 (6.8)	21.6 (6.86)	> 0.05
Anger	17.25 (7.81)	16.75 (8.2)	18.08 (7.2)	< **0.05**	18.35 (10.02)	17.73 (7.69)	17.72 (7.08)	15.64 (6.45)	< **0.05**	16.97 (6.94)	17.49 (8.38)	> 0.05	17.88 (9.2)	17.03 (17.19)	> 0.05
Hostility	18.38 (7.63)	18.01 (7.47)	19.04 (7.87)	> 0.05	20.09 (7.54)	18.98 (7.65)	18.23 (7.53)	16.8 (7.56)	< **0.01**	17.58 (7.74)	18.99 (7.54)	< **0.05**	19.19 (7.52)	18.1 (7.68)	> 0.05
Buss–Perry aggression questionnaire	69.82 (21.68)	69.99 (22.67)	69.72 (20.32)	> 0.05	75.64 (23.17)	71.6 (22.44)	70.25 (20.41)	63.5 (19.64)	< **0.001**	67.04 (20.62)	71.78 (22.29)	< **0.05**	71.12 (22.03)	69.37 (21.63)	> 0.05

Bold values indicate statistical significance

Values are reported as mean (SD)

**Table 2:** Aggression and empathy across different kinds of sports. Values are reported as mean (SD)

	Contact or non-contact sport			Team or individual sport		
	Contact sport	Non-contact sport	*p* value (Mann–Whitney test)	Individual sport	Team sport	*p* value (Mann–Whitney test)
Personal distress	13.07 (5.05)	13.89 (5.51)	> 0.05	13.46 (5.21)	12.68 (5.17)	> 0.05
Empathic concern	19.69 (4.39)	20.16 (6.63)	> 0.05	19.84 (4.52)	19.8 (4.22)	> 0.05
Fantasy	14.25 (5.43)	14.84 (5.76)	> 0.05	14.66 (5.54)	13.32 (5.42)	> 0.05
Perspective-taking	15.32 (4.44)	16.97 (4.17)	< **0.001**	15.88 (4.45)	15.55 (4.3)	> 0.05
Interpersonal reactivity index	62.35 (10.92)	65.89 (12.07)	< **0.01**	63.85 (11.44)	61.36 (11)	> 0.05
Reactive aggression	7.02 (3.52)	7.43 (3.71)	> 0.05	7.16 (3.73)	7.1 (2.74)	> 0.05
Proactive aggression	2.46 (2.91)	2.58 (3.15)	> 0.05	2.57 (2.95)	2.14 (3.12)	> 0.05
Reactive Proactive Aggression Questionnaire	9.48 (5.57)	10.02 (6.05)	> 0.05	9.73 (5.87)	9.25 (4.91)	> 0.05
Verbal aggression	12.58 (4.9)	12.39 (4.7)	> 0.05	12.65 (4.91)	11.89 (4.4)	> 0.05
Physical aggression	21.71 (6.86)	21.37 (6.48)	> 0.05	21.71 (6.88)	21.1 (6.03)	> 0.05
Anger	16.66 (6.74)	17.7 (7.48)	> 0.05	17.4 (7.23)	14.93 (5.16)	> 0.05
Hostility	17.94 (7.4)	19.26 (7.7)	> 0.05	18.61 (7.49)	17.03 (7.52)	> 0.05
Buss–Perry aggression questionnaire	68.91 (20.63)	70.74 (21.6)	> 0.05	70.38 (21.34)	64.97 (18.21)	> 0.05

Bold values indicate statistical significance

### Measures

#### Empathy

The Interpersonal Reactivity Index (IRI) [39] is a 28-item questionnaire answered on a 5-point Likert scale ranging from 0 (does not describe me well) to 4 (describes me very well). The measure has four subscales, and each has seven items: personal distress, empathic concern, fantasy, and perspective-taking. Empathic concern and personal distress measure affective empathy. While fantasy and perspective-taking measure cognitive empathy. The total and each subscale’s scores were calculated by summing all related item scores, and higher scores indicated more empathy, except for nine items, which should be scored reversely [39–41]. We used the Persian version of the interpersonal reactivity index, a reliable (Cronbach’s alpha = 0.79) and valid tool for evaluating empathy in the Iranian population [42].

#### Aggression

The Reactive Proactive Aggression Questionnaire (RPQ) is a self-report questionnaire with 23 questions that measure aggression. This tool assesses adults’ reactive aggression (11 items) and proactive aggression (12 items). Questions are scored on a 3-point Likert scale (never, sometimes, and often) from 0 to 2, in which higher scores indicate higher levels of aggression. Total and subscales’ scores are the sum of all related items’ scores. We used the Persian version of RPQ in our study. Convergent validity and internal consistency (Cronbach’s alpha of 0.85 in males and 0.83 in females) of the Persian version of RPQ have been demonstrated in previous studies [43–45].

The Buss–Perry aggression questionnaire is a 29-item questionnaire that measures four subscales: verbal aggression (5 items), physical aggression (9 items), anger (7 items), and hostility (8 items). This questionnaire is rated on a 5-points Likert scale from 1 (does not apply in my case) to 5 (completely true in my case). Total and each subscale’s scores are the sum of all related items’ scores. We used the Persian version of the Buss–Perry aggression questionnaire in our study, which has its internal consistency (Cronbach’s alpha of higher than 0.7 for all subscales), test-retest reliability, concurrent validity, and known group validity been demonstrated in previous studies [46–48].

### Statistical analysis

We calculated the mean and standard deviation (SD) for continuous variables and the number and percentage for categorical variables. We used the Kolmogorov-Smirnov test to determine whether the continuous variables, including age, IRI, RPQ, and Buss–Perry aggression questionnaires and their subscales’ scores, are distributed normally or not. As none of the variables were distributed normally, we used non-parametric tests, including Mann-Whitney and Kruskal Wallis tests, to compare these variables across groups. We used Pearson’s correlation test to evaluate the association between empathy and aggression subscales. Correlation coefficients were interpreted as follows: 0.1–0.39: weak correlation; 0.4–0.69: moderate correlation, 0.7–0.89: strong correlation; 0.9–1: very strong correlation [49]. Also, we used multiple stepwise linear regressions to determine the factors independently associated with aggression questionnaires’ subscales. We used SPSS version 22 for data analysis. *p* value ≤ 0.05 was considered statistically significant. We performed a posthoc power analysis using G*Power version 3.1.9.7 [50, 51].

## Results

The mean IRI score was 63.36 (SD = 11.37) among our participants. Males had significantly lower scores than females in the fantasy (13.56 vs. 15.66; *p* < 0.001), empathic concern (19.11 vs. 20.95; *p* < 0.001), personal distress (11.92 vs. 15.52; *p* < 0.001) subscales, and total IRI score (60.23 vs. 68.28; *p* < 0.001). Also, 21 to 25 years old participants had significantly lower scores in the empathic concern subscale than older participants (*p* < 0.05). Participants who were 17 to 20 years old had significantly lower scores in the perspective-taking subscale than those older than 30 years (15.19 vs. 16.85; *p* < 0.05). Perspective-taking subscale score and IRI total score were significantly higher in athletes who played non-contact sports than those who played contact sports (*p* < 0.05). There were no other significant differences between demographic groups regarding their IRI and its subscales’ scores (*p* > 0.05).

The mean RPQ score was 9.73 (SD = 5.83) among our participants. Males had significantly higher scores than females in the proactive aggression subscale (2.94 vs. 1.92; *p* < 0.01). Those aged 17 to 20 years had significantly higher proactive and reactive aggression subscales and RPQ total scores than those aged 26 years or older (*p* < 0.05). Also, participants who were 21 to 25 years had significantly higher scores in the reactive aggression subscales and RPQ total score than those older than 30 years (*p* < 0.05). Married participants had lower scores across all RPQ subscales and their total scores than unmarried participants (*p* < 0.05). There were no other significant differences between groups regarding the RPQ and its subscales’ scores (*p* > 0.05).

The mean total score of the Buss–Perry aggression questionnaire was 69.82 (SD = 21.68) among our participants. Males had significantly higher scores than females in the physical aggression subscale (22.5 vs. 20.44; *p* < 0.01); however, females had higher scores in the anger subscale (18.08 vs. 16.75; *p* < 0.05). Participants older than 30 years had significantly lower scores than younger participants in the physical aggression subscale score and Buss–Perry aggression questionnaire total score (*p* < 0.05). Participants older than 30 also had significantly lower scores than those aged 17 to 25 in the hostility subscale (*p* < 0.05). The Buss–Perry aggression questionnaire’s total score and its subscales’ scores, except the anger subscale, were significantly higher in unmarried participants than in married ones (*p* < 0.05). There were no other significant differences between groups regarding the Buss–Perry aggression questionnaire total score and subscales’ scores (*p* > 0.05).

The score of the fantasy subscale of the IRI had weak positive correlations with the RPQ and Buss–Perry aggression questionnaire and all their subscales’ scores (*p* < 0.05). The empathic concern subscale score correlated weakly and reversely with RPQ and its proactive subscale scores (*p* < 0.05). It also had weak reverse correlations with the score of the verbal aggression subscale of the Buss–Perry aggression questionnaire (*p* < 0.05). The score of the perspective-taking subscale of the IRI had weak reverse correlations with the RPQ and Buss–Perry aggression questionnaire and all their subscales’ scores (*p* < 0.05). The score of the personal distress subscale of the IRI had weak positive correlations with RPQ and Buss–Perry aggression questionnaire and all their subscales’ scores (*p* < 0.05), except with proactive aggression score. IRI total score had a weak positive correlation with the score of the reactive aggression subscale of RPQ (*p* < 0.05). It also had weak positive correlations (*p* < 0.05) with the scores of the anger and hostility subscales of the Buss–Perry aggression questionnaire (Table 3).

**Table 3:** Correlation between empathy and aggression in athletes

	Empathic concern	Perspective-taking	Personal distress	Interpersonal reactivity index	Proactive aggression	Reactive aggression	Reactive Proactive Aggression Questionnaire	Physical aggression	Verbal aggression	Anger	Hostility	Buss–Perry aggression questionnaire
Fantasy	0.16***	0.001	0.309***	0.693***	0.095*	0.195***	0.173***	0.095*	0.122**	0.147**	0.244***	0.191***
Empathic concern		0.244***	0.139**	0.619***	− 0.241***	− 0.065	− 0.161***	− 0.092*	− 0.098*	− 0.011	0.034	− 0.038
Perspective-taking			− 0.191***	0.397***	− 0.23***	− 0.341***	0.266***	− 0.336***	− 0.235***	− 0.318***	− 0.281***	− 0.37***
Personal distress				0.576***	0.086	0.353***	0.266***	0.161***	0.169***	0.385***	0.362***	0.351***
Interpersonal reactivity index					− 0.9	0.094*	0.014	− 0.058	0.004	0.113*	0.178***	0.085
Proactive aggression						0.526***	0.848***	0.437***	0.247***	0.288***	0.234***	0.387***
Reactive aggression							0.897***	0.579***	0.376***	0.508***	0.45***	0.609***
Reactive Proactive Aggression Questionnaire								0.589***	0.36***	0.495***	0.403***	0.588***
Physical aggression									0.533***	0.55***	0.513***	0.81***
Verbal aggression										0.477***	0.479***	0.728***
Anger											0.532***	0.826***
Hostility												0.811***

*p < 0.05; **p < 0.01; ***p < 0.001

Results of multiple linear regression evaluating the factors independently associated with different kinds of aggression are shown in Tables 4 and 5. The scores of the IRI’s fantasy and personal distress subscales were positively associated with proactive and reactive regression scores (*p* < 0.05). The score of the perspective-taking subscale of IRI was reversely associated with proactive and reactive aggression scores (*p* < 0.05). The score of the empathic concern subscale of IRI had a negative association with the proactive aggression score (*p* < 0.001).

**Table 4:** Results of the multiple linear regression determining the factors that independently predicted RPQ subscales’ scores

Dependent variable	Predictor	Beta (unstandardized)	95% CI for beta	*p* value	R^2^	Power
Proactive aggression	Age	− 0.055	(− 0.089)–(− 0.02)	< **0.01**	0.148	1
	Female gender	− 1.015	(− 1.594)–(− 0.437)	< **0.01**		
	Fantasy	0.066	0.16–0.116	< **0.01**		
	Empathic concern	− 0.126	(− 0.188)–(− 0.065)	< **0.001**		
	Perspective-taking	− 0.087	(− 0.15)–(− 0.024)	< **0.01**		
	Personal distress	0.069	0.012–0.126	< **0.05**		
Reactive aggression	Age	− 0.091	(− 0.128)–(− 0.054)	< **0.001**	0.249	1
	Contact sport	0.672	0.048–1.296	< **0.05**		
	Fantasy	0.064	0.01–0.117	< **0.05**		
	Perspective-taking	− 0.214	(− 0.281)–(− 0.147)	< **0.001**		
	Personal distress	0.187	0.128–0.246	< **0.001**		

Bold values indicate statistical significance

**Table 5:** The multiple linear regression results determine the factors that independently associated with Buss–Perry aggression questionnaire subscales’ scores

Dependent variable	Predictors	Beta	95% CI for beta	*p* value	R^2^	Power
Physical aggression	Age	− 0.183	(− 0.257)–(− 0.11)	< **0.001**	0.194	1
	Female gender	− 2.4	(− 3.622)–(− 1.179)	< **0.001**		
	Perspective-taking	− 0.405	(− 0.534)–(− 0.276)	< **0.001**		
	Personal distress	0.239	0.122–0.355	< **0.001**		
Verbal aggression	Fantasy	0.109	0.034–0.185	< **0.01**	0.267	1
	Perspective-taking	− 0.257	(− 0.351)–(− 0.162)	< **0.001**		
Anger	Age	− 0.119	(− 0.202)–(− 0.037)	< **0.01**	0.229	1
	Team sport	− 1.955	(− 3.583)–(− 0.327)	< **0.05**		
	Perspective-taking	− 0.414	(− 0.557)–(− 0.271)	< **0.001**		
	Personal distress	0.51	0.389–0.632	< **0.001**		
Hostility	Age	− 0.166	(− 0.246)–(− 0.086)	< **0.001**	0.221	1
	Fantasy	0.195	0.079–0.31	< **0.01**		
	Perspective-taking	− 0.328	(− 0.47)–(− 0.187)	< **0.001**		
	Personal distress	0.418	0.292–0.544	< **0.001**		

Bold values indicate statistical significance

The score of the perspective-taking subscale of IRI had negative associations with all Buss–Perry aggression questionnaire subscales’ scores (*p* < 0.05). The score of the personal distress subscale of IRI had positive associations with all Buss–Perry aggression questionnaire subscales’ scores (*p* < 0.05), except with the verbal aggression subscale score. The fantasy subscale of IRI had a positive association with the score of the hostility subscale of the Buss–Perry questionnaire (*p* = 0.001).

## Discussion

Few studies have been conducted to evaluate the association between aggression and empathy among athletes. Still, to the best of our knowledge, this is the first study evaluating such an association among a group of Iranian athletes. Our major finding is that not all kinds of empathy are negatively related to aggression among athletes. Perspective-taking and empathic concern are negatively related to different kinds of aggression, but on the other hand, personal distress and fantasy had positive associations with aggression.

We found that perspective-taking was negatively related to proactive aggression, reactive aggression, physical aggression, verbal aggression, anger, and hostility. This finding aligns with previous studies, which reported that perspective-taking and empathy could prevent aggression in athletes [11, 26]. In 2012, Stanger et al. found that taking the opponent’s perspective can inhibit aggression in athletes, especially among male athletes. They also found that feeling guilt is a mediator of the association between empathy and aggression, as people with higher empathy experienced more guilt when they acted aggressively. They were also less likely to aggress [11]. In another study, Stanger et al. found that perspective-taking could inhibit aggression in male and female athletes at low provocation; however, at high provocation, perspective-taking could only prevent aggression in females [26]. These studies are in line with our findings as we found that perspective-taking is reversely associated with all types of aggression, including reactive aggression, which was evaluated in the Stanger et al. study [26]. Davis defined perspective-taking in IRI as the “tendency or ability of the respondent to adopt the perspective, or point of view, of other people” [40], which can be considered a cognitive component of empathy [52]. In previous studies on students, affective perspective-taking was more responsible for preventing aggression than cognitive empathy [53]. Ours and Stanger et al.’s [11, 26] findings indicate the possible role of cognitive empathy in preventing aggression in athletes, which may have a stronger role in preventing aggression than in other populations. Interventions to enhance perspective-taking in athletes may effectively prevent aggression considering its role in preventing all types of aggression and similar successful interventions in other populations [54].

One of the differences between our findings and Stanger et al. was that the perspective-taking score had a reverse association with the anger score in our study, which is in contrast to Stanger et al.’s findings, as they did not find any association between empathy and anger [26]. There may be several reasons for such a difference. First, Stanger et al. used Taylor Aggression Paradigm to evaluate aggression, provoked and unprovoked, in a competitive context [55]. In their study, the fictitious opponents used electrical shock for provocation. The authors hypothesized that participants might have felt that the opponents were intentionally hurting them, which may be a reason for reduced empathy toward their opponents [26]. Second, we used self-report questionnaires in our study, and people may act differently in real-world and task-based scenarios. Future studies, especially in real-world situations, are needed to determine the association between anger and perspective-taking in athletes, as there are controversies in this regard.

We found that the score of the personal distress subscale of IRI was positively associated with all types of aggression, except verbal aggression, in contrast to empathy’s inhibitory role in aggression. There are several possible explanations for this finding. First, personal distress has been described as the negative side effect of empathy [56], as not all types of empathy are beneficial [57], and internal distresses may prevent people with higher personal distress scores from empathic interactions [56]. Also, personal distress was found to have associations with neuroticism in previous studies [56], and neuroticism is by itself associated with emotions that often precipitate aggression, such as anger, which can explain the association between personal distress and aggression [58].

The main limitation of this study is that we used self-reported questionnaires as people may have different behaviors than what they report in such questionnaires, considering the role of provocation and arousal in the emergence of aggressive behavior [11, 26]. In addition, these questionnaires are not specifically designed to evaluate athletes and evaluated aggression and empathy in general. Future scenario-based or real-world evaluations are needed to determine the association between empathy better. Also, our study had a cross-sectional design unsuitable for evaluating the causal relationship between variables. Also, we only evaluated Iranian athletes in our study, and there is a need for more studies on aggression and the factors affecting it in athletes from developing countries.

## Conclusion

Perspective-taking is negatively associated with all kinds of aggression in athletes. Future studies can be conducted to determine the possible role of perspective-taking in preventive aggression, which can be a target for interventions. On the other hand, the score of the personal distress subscale of IRI is positively associated with all types of aggression scores, indicating that not all types of empathy inhibit aggression in athletes.

## Data Availability

The datasets generated and/or analysed during the current study are not publicly available due to ensure participants’ confidentiality but are available from the corresponding author on reasonable request.

## Abbreviations

IRI: Interpersonal reactivity index
RPQ: Reactive Proactive Aggression Questionnaire
SD: Standard deviation
